# Performance Evolution and Prediction Model of Dam Polyurethane Insulation Materials Under Multi-Field Coupling Conditions in Hot Summer and Cold Winter Climate Zones

**DOI:** 10.3390/ma18133208

**Published:** 2025-07-07

**Authors:** Lingmin Liao, Hui Liang, Ting Zhao, Wei Han, Yun Dong, Da Zhang, Zhenhua Su

**Affiliations:** 1 Changjiang Scientific Research Institute, Changjiang Water Resources Commission, Wuhan 430010, China; achong17@163.com (H.L.); will_ecust@163.com (W.H.); dongyun@mail.crsri.cn (Y.D.); zhangda@mail.crsri.cn (D.Z.); suzh@mail.crsri.cn (Z.S.); 2 National Research Center on Dam Safety Engineering Technology, Wuhan 430010, China; 3 Research Center of Water Engineering Safety and Disaster Prevention, Ministry of Water Resources, Wuhan 430010, China

**Keywords:** multi-field coupling, hot summer and cold winter climate zones, dam thermal insulation, rigid foamed polyurethane, thermal conductivity, prediction model

## Abstract

This study evaluates the performance degradation of spray rigid polyurethane foam (RPUF) insulation on reservoir dam structures under multi-physics coupling conditions. Focusing on characteristic environmental exposures in Hot Summer and Cold Winter (HSCW) climate zones, accelerated aging tests simulating coupled temperature–humidity effects were conducted to comparatively analyze the thermal resistance and durability evolution between unprotected and encapsulated RPUF configurations. Scanning electron microscopy (SEM), infrared spectroscopy (IR), and other methods were used to characterize and analyze the structure of RPUF. Research has shown that in HSCW climate zones, the thermal conductivity of RPUF gradually increases with the number of degradation cycles, and the insulation performance decreases, mainly due to the damage of the pore structure caused by temperature aging and the combined effect of moisture absorption aging. In comparison, the RPUF after protection can effectively slow down the rate and degree of decline of its insulation performance. On this basis, a time-varying prediction model for the thermal conductivity of RPUF under long-term service in HSCW climate environments was fitted, providing a scientific basis for the durability evaluation of reservoir dam insulation.

## 1. Introduction

Spray rigid polyurethane foam (RPUF), characterized by low thermal conductivity, minimal density, and low water absorption, has been extensively adopted in thermal regulation and protection systems for concrete dam structures [1,2]. Its implementation effectively reduces thermal loads on concrete dam bodies, optimizes stress distribution, and enhances crack resistance and durability. Typical dam insulation systems predominantly employ a composite configuration of “spray polyurethane (PU) base layer with surface protective coating”, where the thermal insulation performance and durability of the RPUF material constitute critical determinants of overall system efficacy. During prolonged service, RPUF undergoes performance degradation due to aging mechanisms induced by multi-factor coupling effects, including thermal cycling, moisture penetration, and UV exposure, thereby compromising material reliability and operational effectiveness [3,4]. Notably, compared to conventional civil and architectural exterior insulation scenarios, reservoir dams are subjected to more complex and severe multi-environmental coupling conditions, rendering the durability of dam surface insulation materials an increasingly prominent concern in both engineering practice and academic research. Consequently, a systematic investigation into the performance evolution mechanisms and degradation prediction modeling of dam RPUF insulation materials holds substantial scientific and engineering significance. Such studies enable rational assessment of insulation system service life and provide technical foundations for ensuring the long-term operational reliability of thermal regulation and protection systems in critical water-retaining structures.

In recent years, scholarly investigations into the performance evolution of RPUF insulation across diverse application scenarios have progressively advanced within domestic and international research communities. Some research groups have implemented isothermal aging protocols on sectioned specimens to methodically characterize the temporal variation in thermal conductivity in medium-density RPUF (about 35.4 kg/m^3^) under controlled aging durations [5,6,7], The aging evaluation of thermal insulation performance for RPUF used in pre-insulated pipes is primarily conducted in accordance with the accelerated aging method prescribed by Standard EN 253 [8] 150-day thermal conditioning at 90 °C. Winkler-Skalna et al. quantitatively analyzed thermal conductivity modifications in RPUF (about 40~120 kg/m^3^) subjected to thermal-oxidative aging at 70 °C [9]. Concurrent investigations by Tian [10] and Alvey [11] have, respectively, elucidated the hygrothermal aging mechanisms under variable temperature–humidity conditions on the insulation efficacy and chemical stability of RPUF, revealing progressive chain scission in polymeric structures and degradation of cellular morphology with extended hygrothermal exposure, which collectively contribute to insulation performance deterioration. Boubakri et al. [4,12,13] systematically investigated the synergistic impacts of hygrothermal aging, UV degradation, and thermal cycling on the thermomechanical properties of thermoplastic PU materials, identifying the synergistic interaction between thermal activation and moisture permeation as the predominant degradation pathway. The hydrolysis aging of PU primarily arises from the hydrolysis of carbamate and urea groups within its molecular chains, with ester linkage hydrolysis being the dominant mechanism. This process generates carboxylic acid, which in turn catalyzes and accelerates further hydrolysis. Concurrently, water molecules diffuse into the polymer network, weakening hydrogen bonding between chains and exerting a plasticizing effect that degrades material properties. Research on the degradation of foamed insulation materials under freeze–thaw cycles identifies water ingress and the subsequent phase change and expansion of water as the primary cause of microstructural damage [14]. Specifically, when water trapped within the pore structure freezes, its volume expansion generates significant frost heave stress, leading to deformation or the rupturing of cell walls, manifested as cell collapse, pore enlargement, and crack propagation—changes that severely compromise the material’s thermal insulation performance.

However, current experimental frameworks predominantly focus on single-factor aging mechanisms, neglecting the critical multi-field coupling effects inherent to actual service environments. Furthermore, the adoption of constant temperature/humidity parameters in these protocols fails to simulate the dynamic environmental loads encountered in practical applications, thereby limiting the fidelity of material degradation simulations [15]. This methodological limitation compromises the accuracy of insulation performance assessment and impedes reliable service life prediction, underscoring the imperative for developing advanced multi-physics coupled aging models that authentically replicate in-service degradation processes.

The performance degradation of insulation materials under operational conditions results from the synergistic effects of multiple environmental factors under prolonged and complex service scenarios. Considering China’s geographical distribution, four representative climatic zones are classified: Hot Summer–Warm Winter, Hot Summer–Cold Winter, Cold, and Severe Cold regions [16]. The HSCW zone, encompassing 16 provinces/municipalities including Hubei, Hunan, Anhui, Zhejiang, and Jiangxi, is predominantly distributed across the Yangtze River basin and southern regions. Characterized by abundant water resources and dense reservoir dam clusters, this zone presents extreme environmental challenges-high temperature and humidity in summer, coupled with damp, cold conditions in winter. These conditions expose dam insulation layers to significant thermo-hygric cyclic coupling erosion risks, critically impacting long-term service performance. For instance, at Xianghongdian Reservoir in Anhui Province (within this climatic zone), the dam surface insulation system has operated for over a decade, exhibiting partial cracking and spalling failures in the protective mortar layer, with the thermal conductivity of RPUF insulation materials increasing by 22.9% compared to initial values. While recent studies have reported degradation patterns of RPUF insulation in cold climate applications (e.g., roofing and tunnel engineering) [2,17], systematic investigations remain lacking regarding the performance evolution and durability prediction models for dam-applied RPUF insulation under multi-field coupling conditions specific to the HSCW zone.

Addressing this research gap, this study employs accelerated aging tests to elucidate the thermal performance evolution mechanisms of RPUF insulation under multi-physics coupling conditions representative of HSCW climatic stresses. A time-dependent predictive model for thermal conductivity evolution is developed, incorporating critical degradation parameters. The findings provide technical support for durability assessment and long-term maintenance strategies of thermal insulation systems in hydraulic and hydropower infrastructure within analogous climatic regions, ultimately contributing to enhanced operational reliability of dam temperature control systems.

## 2. Experiment

### 2.1. Experimental Materials

In the dam thermal insulation and protection system comprising a spray RPUF layer, rendering mortar layer, and surface finish layer as protective components, the RPUF serves as the primary material providing essential thermal insulation and heat barrier functions for the dam structure. The rendering mortar layer and surface finish layer principally function as protective barriers, delivering critical waterproofing performance and anti-aging protection for the RPUF insulation core. Consequently, the durability of the RPUF insulation material is the critical factor determining the overall effectiveness and longevity of the entire thermal insulation system. To account for actual service conditions and facilitate comparative analysis, this experiment focuses on testing RPUF insulation materials under both exposed and protected states.

**Figure 1:**
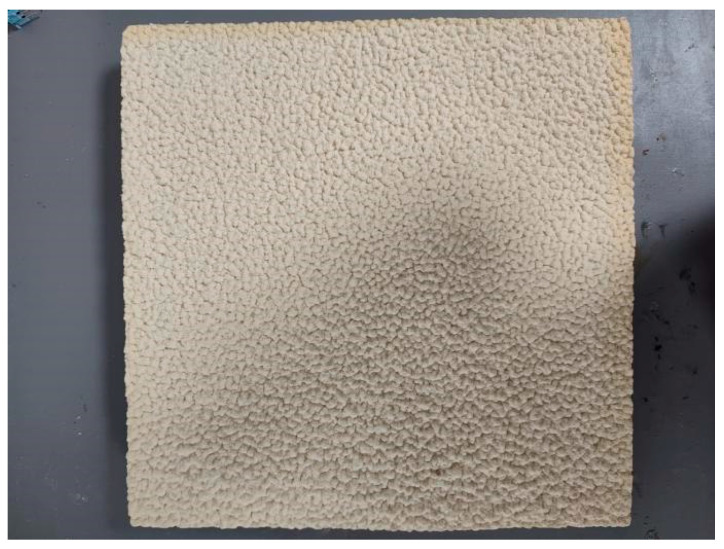
RPUF panels in their original state.

Here, the spray RPUF panels in their original state were produced by Dalian Xiangsheng Polyurethane Co., Ltd. (Dalian, China), and their key performance parameters are summarized in Table 1. Thirty RPUF panels were fabricated using high-pressure airless spraying equipment, through processes of uniform mixing, high-pressure spraying, instantaneous atomization, and on-site foaming, with dimensions of approximately 0.6 m × 0.6 m × 5 cm, as shown in Figure 1. These insulation panels were then precision-cut into test specimens of required dimensions using a foam cutting machine, in strict compliance with the specifications outlined for each performance evaluation. Within this, a total of 96 thermal conductivity test specimens, each about 0.2 m × 0.3 m × 3 cm cut from these original RPUF panels, were used in this study, distributed across 2 statuses (protected and exposed) and 8 aging times, with 3 specimens in a group. A total of 48 specimens were used for aging tests, and another 48 specimens served as control verification.

**Figure 2:**
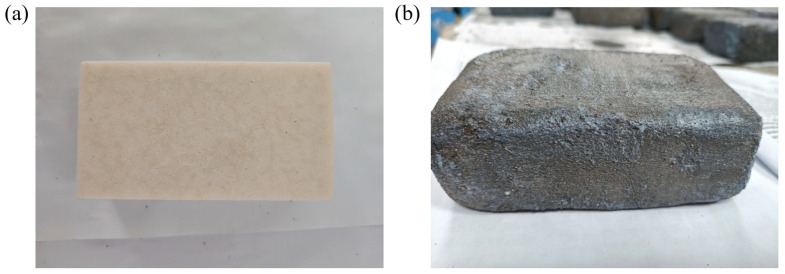
(**a**) Exposed RPUF insulation material specimen; (**b**) protected RPUF insulation material specimen.

RPUF fabricated via spray foaming forms a rough, dense, and hardened skin layer upon curing. While this surface crust exhibits water-resistant properties, its material characteristics differ significantly from the core insulation matrix. To accurately assess the intrinsic performance and engineering behavior of the RPUF, the surface layer was systematically removed. This preparation methodology enables focused investigation of the insulation material’s time-dependent performance degradation patterns under the most adverse simulated service conditions, as shown in Figure 2a.

The preparation process of spray RPUF in protective treatment involves three key steps: firstly, uniform application of a bonding primer onto the RPUF surface; subsequently, layering with a composite waterproof mortar adhesive and embedding acid–alkali resistant mesh fabric to enhance structural stability; finally, coating with a crystalline waterproofing agent to establish a durable protective layer (Figure 2b). The bonding primer, composite waterproof mortar adhesive, and silicone-based crystalline waterproofing agent were supplied by Jinzhai Dongnan Thermal Insulation Materials Co., Ltd. (Lu’an, China). The reinforced alkali-resistant glass fiber mesh fabric was provided by Anhui Jingyu Shidai Chuangzhan Building Materials Co., Ltd. (Hefei, China).

### 2.2. Experimental Methods and Equipment

The HSCW zones of China feature coldest-month average temperature of 0–10 °C (extreme low: −10 °C) and hottest-month average temperature of 25–29 °C (extreme high: 40 °C) [17]. Taking Xianghongdian Reservoir in western Anhui (located in this zone) as an example, it records a coldest-month average of 2.84 °C (extreme low: −13.9 °C), hottest-month average of 27.7 °C (extreme high: 41.4 °C), with relative humidity (RH) ranging 35–95% annually (typically 70–80%), where high humidity coincides with elevated temperatures. This study simulated the regional climate characteristics through accelerated aging tests by amplifying key environmental factors. The simplified parameters included temperature (−10 °C to 40 °C) and humidity (70–95% RH).

A F/GSR 150L temperature–humidity test chamber was employed for cyclic aging tests on RPUF specimens. Each 9 h cycle began at (23 ± 2)°C/50% RH, followed by 0.5 h ramp to 40 °C/95% RH; 5.5 h maintenance at 40 °C/95% RH; 1 h cooling to −10 °C; and 2 h low-temperature exposure. Thermal conductivity was measured every 5 cycles using three-specimen averages.

The temperature–humidity cyclic accelerated aging tests were performed using an F/GSR-150L temperature–humidity cycling test chamber (Shanghai Fengshi Laboratory Instruments Co., Ltd., Shanghai, China), featuring a temperature range of −20 °C to 150 °C and an RH control range of 30% to 98%. The thermal conductivity of RPUF insulation materials was measured using a TC1000 thermal conductivity meter (Xi’an Xiaxi Electronics Technology Co., Ltd., Xi’an, China) based on the plate method. The test conditions were as follows: 25 ± 2 °C, 50 ± 5% RH, a contact pressure of 2–5 kPa, and a duration time of 3–4 h. Microstructural analysis of the PU insulation materials was conducted on a SHIMADZU IRAffinity-1S Fourier transform infrared (FTIR) spectrometer (Kyoto, Japan). The microscopic pore structure and morphology characterization were carried out using a JEOL JSM6610A scanning electron microscope (SEM) (Tokyo, Japan), operated at an acceleration voltage of 20 kV.

A three-month water immersion test was conducted to investigate moisture absorption evolution in RPUF insulation materials, with periodic measurements at designated intervals [18]. 1stopt software (version 1.5) was used for fitting analysis of accelerated aging test data.

## 3. Analysis of Experimental Results

### 3.1. Microstructure and Morphological Characterization of Original Spray RPUF

**Figure 3:**
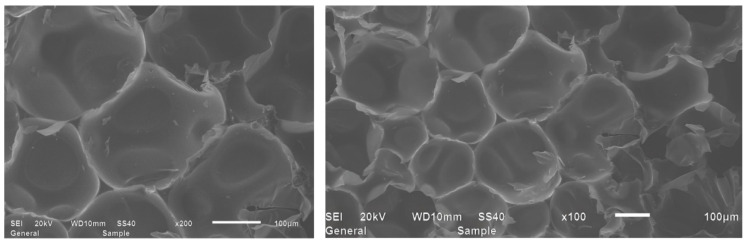
SEM images of the original spray RPUF microstructure.

Figure 3 presents the SEM images of the spray RPUF specimen, revealing its initial surface morphology. As observed, the RPUF exhibits a continuous cellular structure predominantly composed of closed-cell pores. The cellular architecture demonstrates uniform cell size, regular arrangement, intact cell walls, and well-developed cell membranes, with minimal presence of open-cell structures. This optimized pore configuration contributes to its enhanced thermal insulation performance.

**Figure 4:**
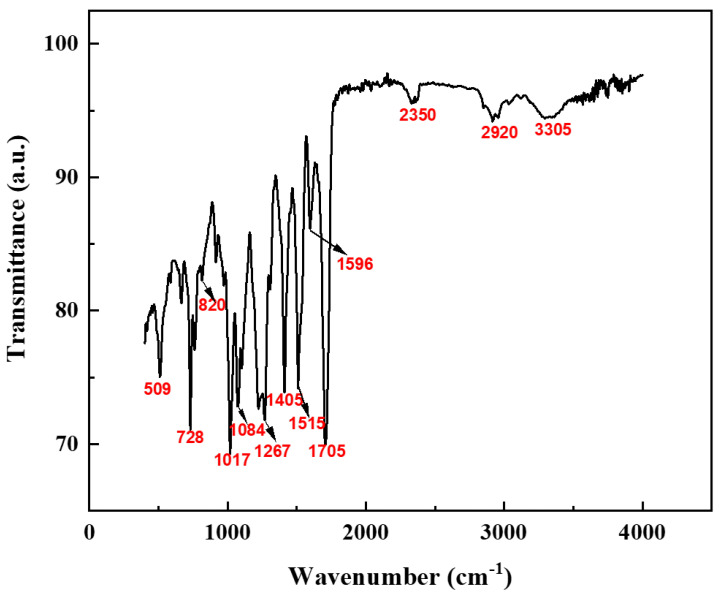
FTIR spectra of the original spray RPUF.

Figure 4 presents the FTIR spectra of the original spray RPUF specimen. It can be observed that the peak at 3305 cm^−^^1^ corresponds to the stretching vibration of the -NH group, the peak at 2920 cm^−^^1^ is attributed to the stretching vibration of saturated C-H bonds, the peak around 2350 cm^−^^1^ is associated with the asymmetric stretching vibration of N=C=O groups, the peak at 1705 cm^−^^1^ corresponds to the stretching absorption of -CONH_2_ and -CONHR groups, the peak at 1596 cm^−^^1^ represents the skeletal absorption of benzene rings, with a corresponding fingerprint peak at 820 cm^−^^1^ indicating para-substitution on the benzene ring, the peak at 1515 cm^−^^1^ is due to the stretching absorption of -NHCO groups, the peak at 1267 cm^−^^1^ corresponds to the stretching absorption of -C-O-C linkages, and the peak at 1084 cm^−^^1^ is attributed to the stretching vibration of C-O bonds.

### 3.2. Water Absorption Characteristics of Original Spray RPUF

**Figure 5:**
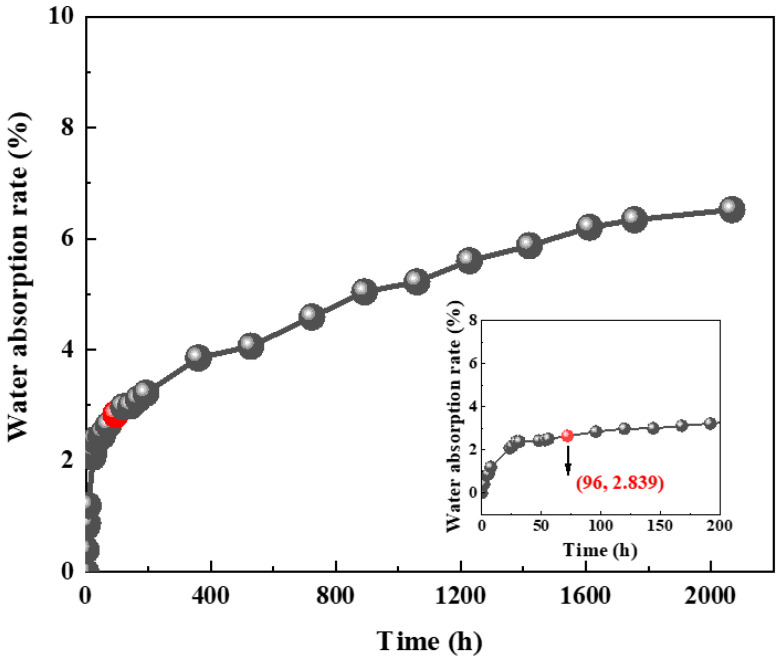
Water absorption versus time for RPUF.

In accordance with DIN 51589 [19], the water absorption of RPUF insulation materials shall not exceed 3% as per the standard 96 h immersion test. This study monitored the water absorption evolution under non-pressurized immersion over approximately three months (Figure 5). The results demonstrate compliance with the specification requirement (<3% absorption after 96 h immersion). However, prolonged immersion revealed continuous water uptake with gradually slowing growth rates, reaching approximately 6.5% after 86 days. Nonlinear curve fitting of the absorption progression indicates conformity to a power function trend, as mathematically expressed in Equation (1), showing high fitting accuracy with a correlation coefficient R^2^ of 0.98.

(1) $$y=a*x^{b}$$

where *x* = service time (h); *y* = water absorption (%); a, b = coefficients. The value of a is 0.7702, and the value of b is 0.2788.

### 3.3. Analysis of Indoor Accelerated Aging Test Results

Both the impact of freeze–thaw cycle temperature fluctuations on the structural aging of RPUF under normal service conditions and the effect of humidity changes on the aging of RPUF’s thermal conductivity were considered in this experimental plan. The aging effect was the coupling of temperature and humidity.

**Figure 6:**
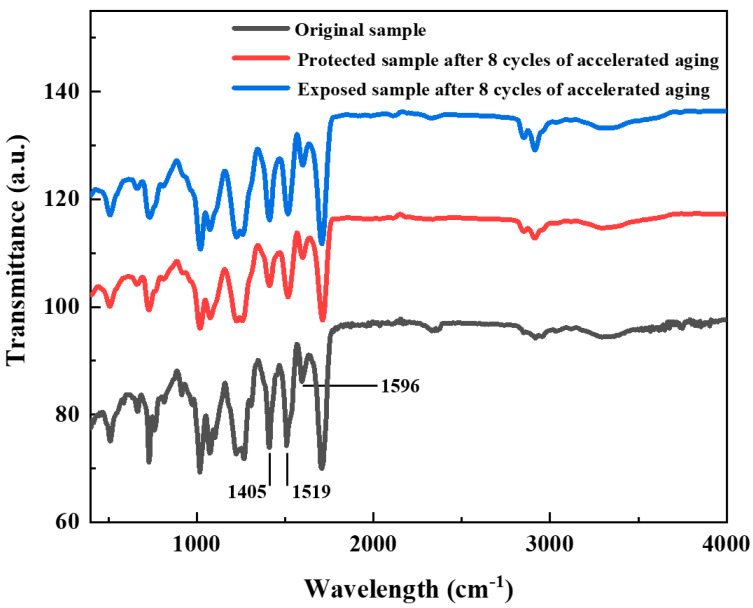
FTIR spectra comparison of RPUF specimens before and after accelerated aging.

Figure 6 shows a comparison of the FTIR spectra of the RPUF specimens before and after accelerated aging. It could be observed that the characteristic peak positions in the FTIR spectra of both the exposed and protected specimens after accelerated aging were largely consistent with those of the original specimens. This indicated that no changes occurred in the types of functional groups in their molecular chain structures.

Since the molecular aging of PU primarily involves hydrolysis of urethane groups, Equation (2) can be employed to characterize the hydrolysis-induced aging degree of PU urethane groups:

(2) $$I=\frac{A_{1519}}{A_{1596}}$$

In the formula: *I*—Urethane index;

*A*_1519_—Peak area of the urethane group absorption band at 1519 cm^−^^1^;

*A*_1596_—Peak area of the benzene ring absorption band at 1596 cm^−^^1^.

Comparative analysis showed that the I value of the original RPUF specimen was approximately 3.28. After eight cycles of accelerated aging, the I value of the protected specimen decreased to about 3.10, while the I value of the exposed specimen decreased to about 2.99. This suggested that hydrolytic aging occurred during the accelerated test [12,13], with the exposed specimens undergoing a relatively higher degree of aging compared to the protected specimens.

**Figure 7:**
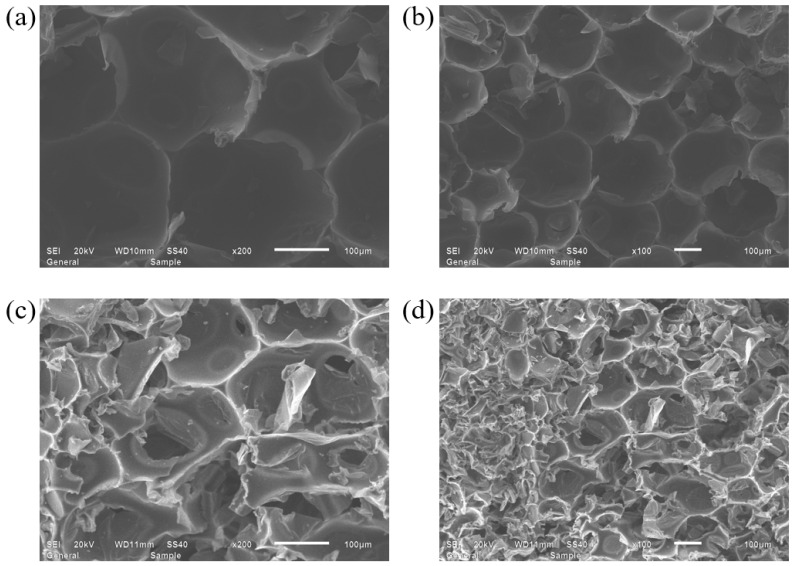
SEM images of (**a**,**b**) protected RPUF specimen after 8 cycles of accelerated aging, (**c**,**d**) exposed RPUF specimen after 8 cycles of accelerated aging.

Figure 7 shows the SEM images of the microstructure of the protected and exposed specimens after eight cycles of accelerated aging under temperature–humidity coupled alternating conditions. It can be observed that after eight cycles, the internal microstructure of the protected specimens largely retained the integrity of the pore framework, with the main changes being an increase in the proportion of open pores and damaged bubble walls compared to the original specimens. In contrast, the exposed specimens showed a significant increase in the proportion of damaged pore structures, with a noticeable increase in the number of broken bubble walls and fractured framework structures. The closed pores with well-preserved bubble walls and framework structures were almost absent. Based on the FTIR results shown in Figure 3 and the comparative analysis, it could be concluded that during the accelerated aging test, the exposed specimens experienced molecular structure aging due to temperature effects and, on the other hand, suffered from moisture absorption, which led to water ingress and frost-induced expansion, resulting in damage to the pore structure. These two factors acted in tandem, accelerating the damage to the bubble walls and the fracture of the framework structure in the RPUF. The protective layer in the protected specimens, however, alleviated both temperature-induced and moisture-induced aging, providing effective waterproofing and anti-aging effects.

**Figure 8:**
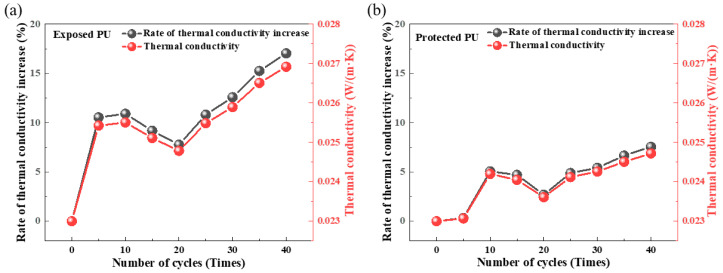
Thermal conductivity evolution of (**a**) exposed RPUF and (**b**) protected RPUF specimens under coupled temperature–humidity cycling.

Figure 8 illustrates the variations in thermal conductivity of exposed and protected RPUF specimens, respectively, under multi-field coupling effects over the number of cycles. Both types of RPUF exhibit similar trends in thermal conductivity changes. After 5 to 10 cycles, a sudden increase in thermal conductivity occurs at a relatively rapid growth rate [17]. This phenomenon is primarily attributed to the presence of a small number of open-cell pore structures within the RPUF insulation material’s cellular structure. When dry, the thermal conductivity of RPUF is significantly lower than that of water or ice after water absorption or even freezing. Initially, as water infiltrates the specimens, the thermal conductivity changes markedly and increases rapidly. However, there is a transient decrease in thermal conductivity between the 10th and 20th cycles, followed by a sustained increase in thermal conductivity as the number of cycles increases beyond 20. At the same number of cycles, the thermal conductivity of the protected RPUF specimens is notably lower than that of the exposed specimens. Taking the 25th and 40th cycles as examples, the thermal conductivity of the protected RPUF specimens increases by 4.8% and 7.5%, respectively, compared to their initial state, whereas the exposed RPUF specimens increase by 10.8% and 17.0%, respectively. This indicates that under multi-field coupling effects of external temperature and humidity, RPUF insulation materials directly exposed to service environments are more prone to aging and experience a faster decline in thermal insulation performance. The presence of a surface protective layer can effectively slow down the growth rate of thermal conductivity in RPUF, resulting in a relatively smaller degree of decline in thermal insulation performance.

### 3.4. Time-Dependent Prediction Model for Thermal Conductivity of RPUF in Different States

The thermal insulation performance of porous materials depends on both the thermal conductivity of the solid skeleton and the fluid within the pores, as well as the spatial structure of the solid skeleton (pore size, shape, and distribution). Therefore, the equivalent thermal conductivity at the macroscopic scale is commonly used to describe this performance. Due to the complex microstructure composed of two or three phases (solid, liquid, gas), theoretical calculations of thermal conductivity typically require simplified models. In most cases, heat conduction—the dominant heat transfer mechanism within porous media—is primarily influenced by the multiphase composition and the heat transfer between phases. For isotropic, heterogeneous porous materials where heat conduction is considered, the series and parallel models are widely applied for calculating equivalent thermal conductivity. The classic parallel model simplifies the structure into multiple parallel cylindrical channels, with heat flow parallel to the channel direction. Each phase (solid, liquid, gas) conducts heat independently, and no heat exchange occurs at the channel interfaces. The series model, conversely, treats the porous material as vertically stacked layers, with heat flow perpendicular to the channels. Heat sequentially passes through each phase, and heat exchange occurs at the interfaces. In comparison, the series model is relatively conservative, while the parallel model better reflects the dominant role of highly conductive phases (solid, liquid) in porous materials. Consequently, the thermal conductivity calculated by the parallel model is generally higher than that obtained from the series model [20,21].

For RPUF with cellular microstructure, the thermal conductivity increase under coupled temperature–humidity exposure originates from dual mechanisms: thermal aging-induced cellular degradation and hygroscopic aging effects.

When moisture-absorbed, water displaces air within the cellular structure of RPUF. This water acts as a thermal bridge, forming mixed heat transfer pathways composed of the solid skeleton, air, and water [22]. Furthermore, under prolonged moisture intrusion, building upon the effects of thermal aging, the degradation of bubble walls and fracture of the skeletal structure are accelerated. Water infiltrates adjacent pores, causing the volume of water-filled cavities to gradually expand. Macroscopically, this manifests as an increase in water absorption rate. That is, the destruction of the porous structure and the increase in water absorption rate form a mutually reinforcing relationship, accelerating the degradation of the material’s insulation performance [23].

Based on the above considerations, since the calculation results of the parallel model are closer to the theoretical upper bound of the thermal conductivity range, this approach will be adopted to thoroughly investigate the impact of the harsh service environment in reservoir dams on the insulation performance of RPUF. Building upon the classical parallel model (Equation (3)) for theoretical prediction of thermal conductivity in porous insulation materials [24], this study develops a quantitative framework correlating structural degradation and moisture absorption with thermal performance evolution under HSCW climate conditions, as mathematically formulated in Equations (4)–(9).

According to the parallel rule, the thermal conductivity of a three-phase porous material can be expressed as

(3) $$\lambda =\lambda_{s}V_{S}+\lambda_{a}V_{V}(1-S_{r})+\lambda_{w\;}V_{V}S_{r}$$

In this equation, *λ_S_* was the thermal conductivity of solid materials; *V_S_* was the volume fraction of solid material; *λ_a_* was the thermal conductivity of gases; *V_V_* was the pore volume fraction; *λ_w_* was the thermal conductivity of water; *S_r_* was water saturation; and *V_S_ + V_V_ =* 1.

During the aging process of RPUF materials under temperature–humidity coupling, the main changed parameters included the moisture saturation degree *S_r_*, the volume fraction of pores *V_V_*, and the thermal conductivity λ. Therefore, at different service timing *t*_1_ and *t*_2_, corresponding values of *S_r_*_1_ and *V_V_*_1_ and *λ*_1_, *S_r_*_2_, *V_V_*_2_, and *λ*_2_ could be obtained. Substituting these boundary conditions into Equation (1) and rearranging, we could get the following:

(4) $$\lambda_{2}-\lambda_{1}=(\lambda_{s}-\lambda_{a})(V_{V1}-V_{V2})+(\lambda_{w\;}-\lambda_{a}){(V}_{V2}S_{r2}-V_{V1}S_{r1}$$

Since $V_{V\;}S_{r}$ = $V_{w\;}$, Equation (4) can be simplified to the following:

(5) $$∆\lambda =(\lambda_{s}-\lambda_{a})∆V_{v}+(\lambda_{w\;}-\lambda_{a})∆V_{w}$$

The initial thermal conductivity of RPUF could be recorded as λ_0_, then the growth rate of thermal conductivity at different time points could be expressed as follows:

(6) $$\frac{∆\lambda }{\lambda_{0}}=\frac{\lambda_{s}-\lambda_{a}}{\lambda_{0}}∆V_{v}+\frac{\lambda_{w\;}-\lambda_{a}}{\lambda_{0}}∆V_{w}$$

Because the thermal conductivities of air, water, and RPUF skeleton were fixed, it could be concluded that the change rate of thermal conductivity of RPUF was related to the aging rate of material structure and the moisture absorption rate.

According to the Arrhenius equation, in a certain temperature range, the relationship between the change in material properties and aging time could be expressed by the following equation:

(7) $$∆P=\frac{{P_{0}-P}_{t}}{P_{0}}=1-e^{-kt}$$

where ∆P is the change rate of material properties; *t* indicates the aging time.

The mechanical behavior evolution of RPUF primarily stems from pore structural evolution, establishing a positive correlation between ΔP and $∆V_{v}$ that can be modeled through a negative exponential function. Concurrently, the moisture absorption progression $∆V_{w}$ demonstrates power-law dependence on exposure duration as per Equation (1). Building upon these relationships, nonlinear regression analysis was applied to experimental data from exposed and protected specimens, yielding time-dependent predictive models for thermal conductivity evolution in both material states.

Exposed state:

(8) $$\frac{∆\lambda }{\lambda_{0}}=7.4519\times (1-\exp(-9.2018x))+0.1916x,\;R^{2}=0.9304$$

Protected state:

(9) $$\frac{∆\lambda }{\lambda_{0}}=1637\times (1-\exp(-0.00004x))+0.6797x^{0.5},\;R^{2}=0.9233$$

Here, x was the number of freeze–thaw cycles, and the unit was times. $\frac{∆\lambda }{\lambda_{0}}$ indicates the thermal conductivity growth rate, expressed in %.

**Figure 9:**
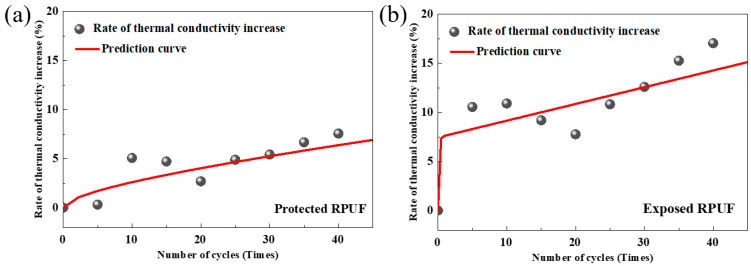
Measured vs. predicted thermal conductivity of (**a**) protected RPUF and (**b**) exposed RPUF.

To validate the predictive capability of the proposed model, Figure 9 compares experimental measurements and model predictions of thermal conductivity for exposed and protected RPUF insulation materials after varying freeze–thaw cycles. The distribution of data points, composed of experimental and predicted values, demonstrates a relatively good linear correlation (r = 1), indicating acceptable deviations between predicted and measured values within engineering tolerance limits. This validation confirms the model’s reliability for estimating thermal conductivity evolution in RPUF insulation subjected to different numbers of freeze–thaw cycles.

According to existing joint research reports from the Tianjin Architectural Design Institute and the Department of Civil Engineering at Tianjin University [25], meteorological data from the official website of the China Meteorological Administration (CMA) were utilized to retrieve historical climate statistics for representative cities, and statistical analysis of freeze–thaw cycle frequencies in these representative cities was conducted. For the HSCW zone (with Nanjing as a representative city), the annual freeze–thaw cycle frequency is approximately six times. By substituting these values into Formulas (7) and (8), it is predicted that the growth rates of the thermal conductivity of RPUF under exposed conditions after 5, 10, and 13 years of service are approximately 13.2%, 18.9%, and 22.4%, respectively, while under well-protected surface conditions, the growth rates are approximately 5.7%, 9.2%, and 11.1%, respectively. Validation was conducted using field-measured data from the Xianghongdian Reservoir [16], located in the HSCW climate zone. The RPUF surface protection layer exhibited localized blistering, reduced crack resistance, and water seepage. After 13 years of service, the thermal conductivity of the insulation material increased by 22.9%. Comparative analysis reveals strong agreement between the predicted thermal conductivity values of unprotected RPUF derived from the computational model and field-measured data. This correlation aligns with observed partial failures in the surface rendering mortar layer, demonstrating that the model accurately simulates material degradation under compromised protective conditions. This indicates that the fitted functional model can better reflect the evolution law of the thermal conductivity performance of RPUF insulation materials for dams in the HSCW zone with respect to service environment and service time.

It should be noted that in establishing the time-dependent thermal conductivity model for RPUF in this study, the primary assumption is that the porous medium is an isotropic continuous medium. This assumption reasonably reflects the uniform cellular structure characteristics of RPUF to a certain extent. This simplified premise also helps reduce computational complexity, making the calculation process more efficient. Simultaneously, the influences of nonlinear heat transfer paths via convection and radiation are neglected, facilitating a focused analysis of the key influencing factors and fundamental patterns of thermal conductivity. These simplifications offer advantages and significance in enhancing the feasibility, engineering application value, and theoretical framework applicability of research predicting the long-term thermal conductivity growth rate of RPUF in reservoir dams located in HSCW regions. They provide a practical and easily applicable methodological reference for actual engineering. Of course, in practical applications, RPUF materials often exhibit a certain degree of anisotropy, and a single parallel model still struggles to fully encompass the complex factors involved in multiphase heat transfer. This may affect prediction accuracy or limit the application scope of this predictive model. In subsequent research, we will endeavor to describe the actual heat transfer process by introducing more refined thermal conductivity models and seek additional long-term field service measurement data from reservoir dams in HSCW regions to continuously verify and optimize the predictive model.

## 4. Conclusions

This study focuses on the typical hydraulic and hydropower engineering service environment in HSCW climate zones. By comprehensively considering temperature and humidity fields, and combining indoor accelerated aging simulation tests with microstructural characterization and performance testing, it reveals the evolution pattern of thermal conductivity in RPUF insulation materials used on dam surfaces. Under multi-field coupling effects in HSCW climate zones, the thermal conductivity coefficient of RPUF gradually increases with freeze–thaw cycles, leading to degraded insulation performance. This deterioration is primarily caused by pore structure damage from thermal aging and moisture absorption aging effects. Compared to exposed RPUF, protective layers such as surface mortar and decorative coatings can effectively mitigate the rate and extent of thermal performance degradation. Based on these findings, a time-dependent prediction model for the thermal conductivity coefficient of RPUF insulation materials in long-term dam service within HSCW climate zones was established through fitting analysis. This research provides a scientific basis and technical support for rational durability evaluation and long-term operation safety management of dam insulation systems.

## Figures and Tables

**Table 1:** Key performance parameters of spray RPUF.

Parameter	Performance Index
Apparent Density (kg/m^3^)	49
Thermal Conductivity (W/(m·K), Mean temperature: 25 °C)	0.023

## Data Availability

The raw data supporting the conclusions of this article will be made available by the authors on request.
